# Sesquiterpene volatile organic compounds (VOCs) are markers of elicitation by sulfated laminarine in grapevine

**DOI:** 10.3389/fpls.2015.00350

**Published:** 2015-05-19

**Authors:** Malik Chalal, Jana B. Winkler, Karine Gourrat, Sophie Trouvelot, Marielle Adrian, Jörg-Peter Schnitzler, Frank Jamois, Xavier Daire

**Affiliations:** ^1^Institut National de la Recherche Agronomique, UMR AgroSup INRA uB 1347 Agroécologie, Pôle IPM – ERL CNRS 6300, Université de BourgogneDijon, France; ^2^Institute of Biochemical Plant Pathology – Research Unit Environmental Simulation, Helmholtz Zentrum MünchenNeuherberg, Germany; ^3^Plate-forme ChemoSens, Centre des Sciences du Goût et de l’Alimentation, UMR6265 CNRS, UMR1324 INRA, Université de BourgogneDijon, France; ^4^Laboratoires GoëmarSaint-Malo, France

**Keywords:** PTR-QMS, SPME-GC-MS, plant defenses, terpenes, methylsalicylate, downy mildew

## Abstract

Inducing resistance in plants by the application of elicitors of defense reactions is an attractive plant protection strategy, particularly for grapevine (*Vitis vinifera*), which is susceptible to severe fungal diseases. Although induced resistance (IR) can be successful under controlled conditions, in most cases, IR is not sufficiently effective for practical disease control under outdoor conditions. Progress in the application of IR requires a better understanding of grapevine defense mechanisms and the ability to monitor defense markers to identify factors, such as physiological and environmental factors, that can impact IR in the vineyard. Volatile organic compounds (VOCs) are well-known plant defense compounds that have received little or no attention to date in the case of grape-pathogen interactions. This prompted us to investigate whether an elicitor, the sulfated laminarin (PS3), actually induces the production of VOCs in grapevine. An online analysis (proton-transfer-reaction quadrupole mass spectrometry) of VOC emissions in dynamic cuvettes and passive sampling in gas-tight bags with solid-phase microextraction-GC-MS under greenhouse conditions showed that PS3 elicited the emission of VOCs. Some of them, such as (*E,E*)-α-farnesene, may be good candidates as biomarkers of elicitor-IR, whereas methyl salicylate appears to be a biomarker of downy mildew infection. A negative correlation between VOC emission and disease severity suggests a positive role of VOCs in grape defense against diseases.

## Introduction

Grapevine (*Vitis vinifera* sp.) is susceptible to severe fungal diseases, such as downy mildew caused by the oomycete *Plasmopara viticola*. Controlling this disease requires many fungicide applications; therefore, induced resistance (IR) by means of elicitors represents an attractive strategy for reducing fungicide use. Elicitors are compounds perceived by plants as danger signals that trigger cascades of signaling events leading to plant defense expression (Garcia-Brugger et al., 2006). Elicitors are found in various biochemical classes, including lipids, proteins, and carbohydrates. Oligosaccharides derived from cell walls of algae or microorganisms are well-known elicitors now referred to as microbial-associated molecular patterns (MAMPs) that are recognized by cognate plant receptors (Jones and Dangl, 2006).

Laminarin, a natural linear β-1,3 glucan oligosaccharide (degree of polymerization approximately equal to 25) extracted from the brown algae *Laminaria digitata*, elicits defense, and induces resistance in different plant species (Klarzynski et al., 2000; Aziz et al., 2003). Sulfated laminarin (PS3), which is obtained by the chemical sulfation of native laminarin, has been shown to be a much more effective inducer of resistance than the native compound (Ménard et al., 2004). In grapevine, PS3 elicits the expression of defense genes, callose deposition, phytoalexin (resveratrol and derivatives) production, and IR against *P. viticola* (Trouvelot et al., 2008).

Phenolic compounds, particularly the phytoalexin resveratrol and its derivatives, have been extensively studied as functional grapevine defense metabolites (Chong et al., 2009). However, little is known regarding other classes of defense compounds in this species. Recent transcriptomic analyses indicated that treatment with PS3 can upregulate grapevine genes annotated as terpene synthases (Daire, unpublished), suggesting that the production of volatile organic compounds (VOCs) could be triggered by this elicitor.

Volatile organic compounds are secondary metabolites, including terpenes, green-leaf volatiles, and benzoids, emitted from above and belowground plant organs. They exert long- and short-distance effects on different plant-interacting organisms (Dudareva et al., 2006). Among their diverse roles, these compounds are essential components of the plant’s defense repertoire. They can act as repellants of herbivore insects or attractants to their enemies (Dicke et al., 2009), and as airborne defense signals, they can prime defense responses against pathogens in distant plants (Kishimoto et al., 2006; Yi et al., 2009).

The aim of this study was to analyze whether PS3 elicits VOC production. Proton-transfer-reaction quadrupole mass spectrometry (PTR-QMS) and solid-phase microextraction (SPME)-GC-MS were used to address the temporal profile of this emission and its metabolic composition. We further aimed to test whether the induced VOCs can be considered defense biomarkers of grapevine and to gain insights into a possible role of VOCs in grape defense against *P. viticola*.

## Materials and Methods

### Plant Material

Grapevine (*V. vinifera* L. cv. Marselan) herbaceous cuttings were grown in individual pots (10 cm × 7 cm × 7 cm) containing a mixture of peat and perlite (4:1, vol/vol) in a greenhouse with a daytime temperature of 23 ± 4°C and a nighttime temperature of 18 ± 7°C until they developed six to eight leaves (*ca.* 7 weeks). The plants were watered every day with a fertilization solution (0.25% Topfert2 solution NPK 10-10-10+ oligonutrients, Plantin, France).

### Elicitor Treatment and Pathogen Inoculation

Sulfated laminarin (PS3, provided by Goëmar laboratories) was prepared at a concentration of 2.5 mg ml^-1^ in distilled water either with or without 0.02% surfactant (an ethoxylated fatty alcohol provided by Goëmar) and applied to both the upper and lower leaf surfaces using a hand-held sprayer. Water or the surfactant alone was also sprayed as controls according to the experimental design.

The plants were maintained in a greenhouse under the conditions described above. *P. viticola* (Berk. & Curt. ex de Bary). Berl. and de Toni inoculation was performed post-elicitor treatment by spraying a sporangia suspension at a concentration of 10^4^ sporangia per ml as previously described (Trouvelot et al., 2008). For mock-inoculation, which was used as a control, the leaf was sprayed with distilled water.

The disease severity was determined through visual assessment of the leaf surface area covered by sporulation. The disease reduction rate was calculated as [1– (treated sporulating area/control sporulating area)] × 100.

### Online Analysis of VOC Emissions by Proton-Transfer-Reaction Quadrupole Mass Spectrometry

For the online analysis of VOC emissions, a system consisting of two cuvettes made of Perspex glass (volume = 4.5 L), each of which contained one plant, was operated in parallel. Details of the system were previously described (Ghirardo et al., 2012). Synthetic VOC-free air (21% v/v O_2_ in N_2_, 400 ppm CO_2_) was humidified and passed through the cuvettes at a flow rate of 1 l min^-1^. The temperature in the cuvette was approximately 25°C at night and 30°C during the day, the relative humidity was 80–90%, and the day/night cycle was 16/8 h. Irradiation was applied by fluorescent tubes with a photosynthetically active photon flux density (PPFD) of approximately 90 μmol m^-2^ s^-1^ at the canopy level.

The plants were subjected to four different treatments performed at 6 p.m.: water-control-mock-inoculated treatment, surfactant-inoculated treatment, PS3-plus-surfactant-mock-inoculated treatment, and PS3-plus-surfactant-inoculated treatment.

For each experiment, two grapevine plants were transferred from the greenhouse to the laboratory, and the leaves were sprayed with PS3 solution, surfactant, or water. The aboveground parts of the plants were then enclosed in the cuvettes. The leaves were mock- or *P. viticola*-inoculated 1 day post treatment (dpt). The dynamics of the emissions were followed online through PTR-QMS, PTR-QMS500, Ionicon, Austria) for 4 dpt. To enable a successful inoculation 1 dpt, the air flow through the cuvettes was interrupted for 2 h, and light was switched off to improve the conditions for infection.

The calibration of the PTR-QMS instrument, the determination of background signals and the monitoring of the protonated masses were performed as previously described (Ghirardo et al., 2010, 2011).

Three biological replicates were analyzed for each treatment. The leaf area enclosed in the cuvette was determined from individual leaf photographs taken at the start of each experiment using Sigma Scan Pro5 (Jandel Scientific, USA). The final total leaf area at 4 dpt was determined from the growth rates of reference plants placed under comparable light and temperature conditions next to the cuvettes.

Immediately after removing the plants from the cuvettes, leaves two and three (fully expanded, counted from the apex) were placed under humid conditions to provoke sporulation, and the infection rates were determined visually at 6 days post inoculation (dpi) as described above.

### Passive Sampling of Grapevine VOCs by SPME-GC-MS Analysis

The VOCs from grapevine plants were collected using fibers coated with divinylbenzene (DVB), carboxen (CAR), and polydimethylsiloxane (PDMS; StableFlex 1 cm – 50/30 μm, Supelco, Bellefonte, PA, USA). The fibers were conditioned in the gas chromatograph (GC) injector according to the manufacturer’s instructions.

Volatile organic compound sampling was performed at the greenhouse ambient temperature as follows: at 11 a.m., the aboveground parts of each plant were individually bagged in a commercial oven plastic bag (Albal, France). After 1 h of equilibration, SPME fibers were inserted into the top of the bag. They were removed 3 h later, in the same time than the bag. The exposure time adopted for SPME adsorption was selected according to preliminary experimental tests. The same plants were used from the beginning to the end of an experiment.

**FIGURE 1:**
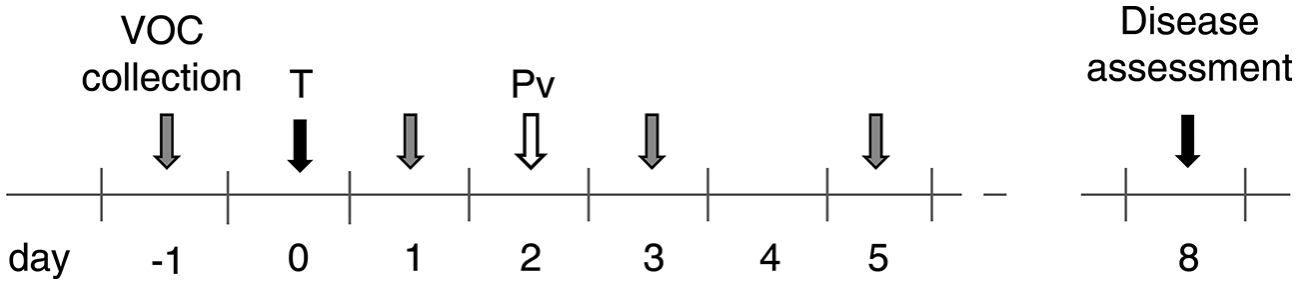
**Outline of treatments and volatile organic compound (VOC) collection times in solid-phase microextraction (SPME) experiments.** The samples were treated with water as a control or PS3 (noted T, day 0, black arrow) and inoculated with *Plasmopara viticola* at 2 dpt (Pv, white arrow). The gray arrows indicate the times of VOC collection (-1, 1, 3, and 5 days with respect to the treatment time). Disease assessment was performed at 8 dpt (black arrow).

The plants were either treated with water or PS3 without surfactant and inoculated 2 days later. After inoculation, the plants were placed overnight in a humid chamber (relative humidity of 100%), and then transferred back to the greenhouse. Sporulation was provoked 6 dpi as described above. The treatments and collection times are depicted in **Figure 1**. Five plants (replicates) were used per condition. The experiments were repeated three times unless otherwise indicated. The leaf surface of each plant was determined at the beginning and end of the experiment from individual leaf photographs using the Image J software.

### Analysis of VOCs Collected with SPME Fibers

An Agilent 6890 GC system coupled with an MSD 5973 mass detector (Agilent Technologies, Palo Alto, CA, USA) was used to separate and detect VOCs. The SPME fibers were desorbed into the GC split/splitless injector at 270°C in the splitless mode for 3 min. After desorption, each fiber was maintained in the injector for 10 min to eliminate any compound from the fiber. The injector and detector temperatures were set to 270°C.

Helium was used as the carrier gas at a constant flow of 1.4 ml min^-1^ with a linear velocity of 44 cm s^-1^. VOCs were separated using a DB-5 MS column (Agilent J&W, 30 m, 0.25 mm i.d., with a phase thickness of 0.5 μm).

The GC oven temperature was programmed as follows: maintained at 40°C for 2 min, increased at a rate of 6°C min^-1^ to 80°C, maintained for 3 min, and increased at a rate of 3.4°C min^-1^ to 170°C and then at a rate of 12°C min^-1^ to 300°C.

The mass spectrometer was used in the electron impact mode (EI) with an ionization energy of 70 eV. The ion source temperature was set to 230°C, the quadrupole temperature was set to 150°C, and the transfer line temperature was set to 280°C. The MS detector was set in the full scan mode in the mass range of 29–300 *m/z*.

Each compound was identified according to its retention index, mass spectrum, and the calculation and comparison of the GC retention index of a series of alkanes (C8–C30) with the retention indexes in published data calculated under the same conditions. Three mass spectra databases were used for comparisons: NIST08 library, Inramass library, and Wiley 138 database. The available VOCs (provided by the INRA aroma library) were injected as standards, and their retention times, and indexes were compared to those of the SPME samples.

The total emission of each compound was calculated and expressed as cumulative histograms of the peak areas (in arbitrary units) normalized to the leaf area.

### Statistical Analyses

Student *t*-test and Pearson correlation coefficient were calculated using the Past software (University of Oslo). Kruskal–Wallis One Way Analysis of Variance on Ranks was performed for the analysis of the PTR-QMS emission rates during the last 120 min using the statistical package of SigmaPlot Version12 (Systat software, Inc., USA).

## Results and Discussion

### PTR-QMs Monitoring of Grapevine Emissions

**FIGURE 2:**
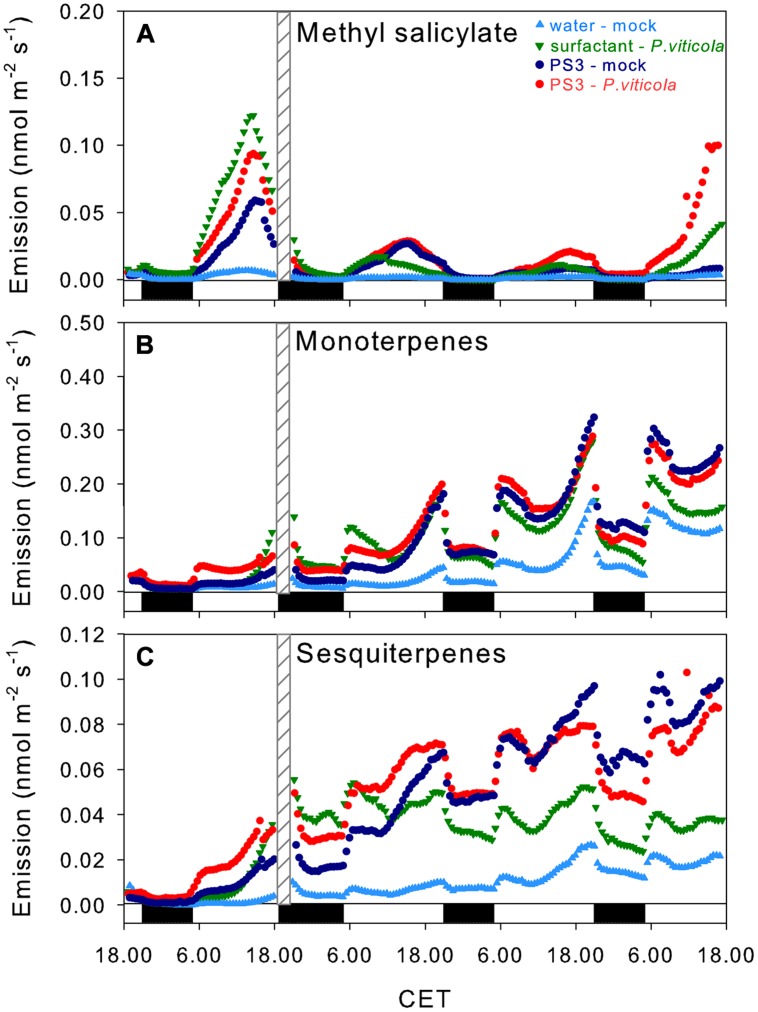
**Emission rates of methyl salicylate **(A)** and mono- **(B)**, and sesquiterpenes **(C)** measured through proton-transfer-reaction quadrupole mass spectrometry (PTR-QMS) during the time course of the experiments.** The values are the means from three biological replicates. The treatments are represented by different colors (light blue: mock-water control, green: surfactant-*P. viticola* inoculated, dark blue: PS3 – mock, and red: PS3 – *P. viticola* inoculated). The day/night cycle is indicated by white and black bars in the graphs. The gray patterned bars represent the time of the day at which inoculation or mock spraying was performed. CET, central European time.

**FIGURE 3:**
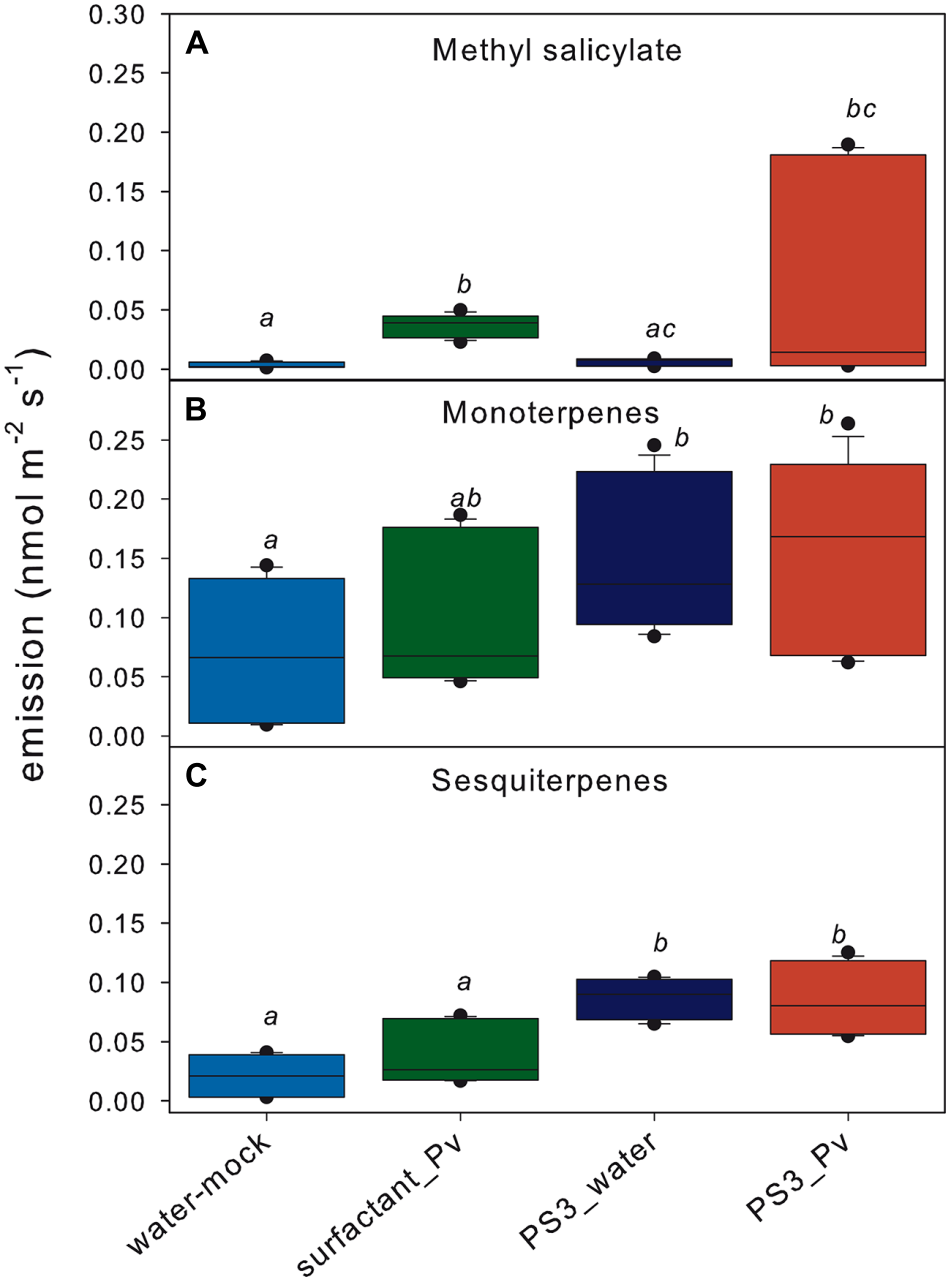
**Mean emission rates of methyl salicylate **(A)**, monoterpenes **(B)**, and sesquiterpenes **(C)** at the end of the time-course study (final 120 min, 4 dpt) obtained through PTR-QMS online measurements.** Each box represents the last five data points for each treatment (three biological replicates per treatment). The treatments with the same letter are not significantly different.

Volatile organic compound emissions, particularly methyl salicylate (MeSA; m/z^+^ 153) and the sums of monoterpenes (m/z^+^ 137) and sesquiterpenes (m/z^+^ 205), were first analyzed dynamically for 4 dpt by PTR-QMS. The monitoring revealed an initial rapid induction of MeSA, which peaked during the light phase 20 h post-treatment (hpt), in all of the treatments containing surfactant (**Figure 2A**). This initial peak of the MeSA emissions decreased sharply, and the level remained low during the following 2 days. The emission of MeSA started to increase again at 4 dpt only in the *P. viticola*-inoculated plants. The emission rates recorded over the last 120 min of the online monitoring period were higher in the inoculated plants than in the mock-inoculated plants (**Figure 3A**), though this difference was only significant for the surfactant-treated inoculated samples, due to high variability of emissions in PS3-treated and inoculated samples.

The first peak of MeSA emission was likely induced by the surfactant, which is consistent with the surfactant-induced up-regulation of a grapevine salicylate *O*-methyltransferase gene (Daire, unpublished). This elicitation can be interpreted as a result of perturbation in the cytoplasmic membrane. Indeed, MeSA is known as a response to various abiotic stressors that disturb the plasma membrane, e.g., wounding and pore-forming toxins (Chen et al., 2003).

The second increase is apparently linked to *P. viticola* infection. This is in agreement with the finding that MeSA is often produced by plants during exposure to biotic stress (Jansen et al., 2011).

The overall emission of monoterpenes (m/z^+^ 137) showed a typical day/night rhythm (**Figure 2B**). This is a feature of leaves without storage pools, where monoterpene emissions are closely related to photosynthetic activity (Ghirardo et al., 2010). The monoterpene emission rates increased during the time course of the experiment in all of the samples and treatments. Nevertheless, the emission rate was slightly higher in the PS3-treated samples (both the inoculated and mock-inoculated samples). At the end of the monitoring period (4 dpt), the monoterpene emission rates were significantly (*p* < 0.05) higher in the PS3-treated samples (**Figure 3B**) compared with the mock-inoculated control. Inoculation with *P. viticola* did not affect the monoterpene emission rates.

Similar to monoterpenes, the total emission of sesquiterpenes (monitored at m/z^+^ 205) displayed a diurnal course with maximum rates close to the end of the light phase (**Figure 2C**). The day-to-day trend of the sesquiterpene emissions was also comparable to that found for the monoterpenes. However, during the final 120 min at 4 dpt, the emission rates of the PS3-treated samples were significantly higher compared with those of the mock-inoculated or surfactant-treated samples, irrespective of *P. viticola* inoculation (**Figure 3C**).

PS3 actually IR in the plants. Assessment of the infection rates at the end of the experiment demonstrated that the application of PS3 reduced the disease level in the experiments (5–25% infected leaf area) compared with the surfactant control (25–85% infected leaf area).

### SPME-GC-MS Analysis of Grapevine Emissions

Because the PTR-QMS analyses were restricted regarding the number of plants and the duration of the experiments, additional experiments were conducted with plants enclosed in static bags and with the passive sampling of VOCs using SPME and subsequent GC-MS to identify the mono- and sesquiterpenes and confirm the induction of MeSA.

In routine IR experiments, surfactant is added to PS3 to facilitate the penetration of the oligosaccharide through the cuticle and therefore improve the level of IR (Daire, unpublished results). However, because PTR-QMS analyses have revealed that this surfactant induces VOCs on its own, particularly MeSA, it was not used in the following SPME experiments to study the specific effect of PS3. Instead, water was used as a control.

Solid-phase microextraction sampling and GC-MS analysis allowed the identification of 47 compounds, which are listed in **Table 1**. These included 18 monoterpenes, one monoterpene alcohol, 24 sesquiterpenes, one sesquiterpene alcohol, one GLV (breakdown product of unsaturated lipid oxidation), MeSA and MeJA. Eighteen terpenes out of the 42 detected were emitted at a higher rate in the PS3-treated samples than in the water control in at least one time point during the course of the experiments. No VOC emission was down-regulated by PS3.

**Table 1:** Grapevine volatile organic compounds (VOCs) identified using solid-phase microextraction (SPME)-GC-MS.

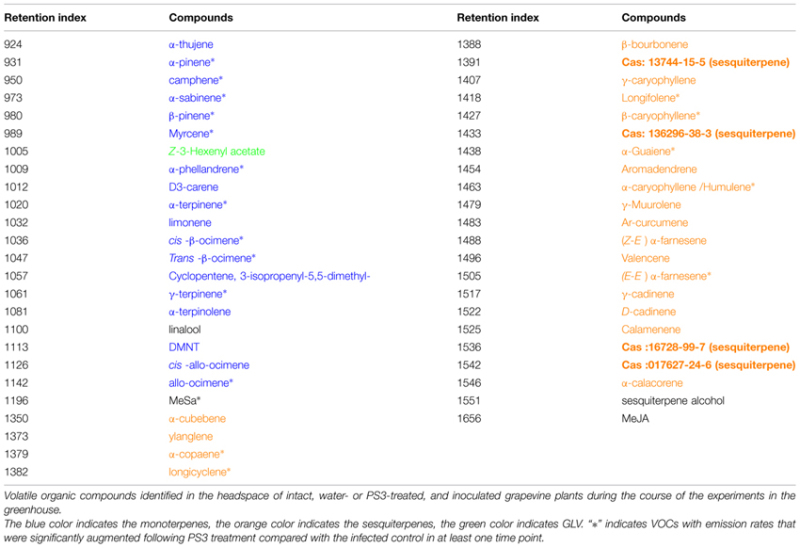

**FIGURE 4:**
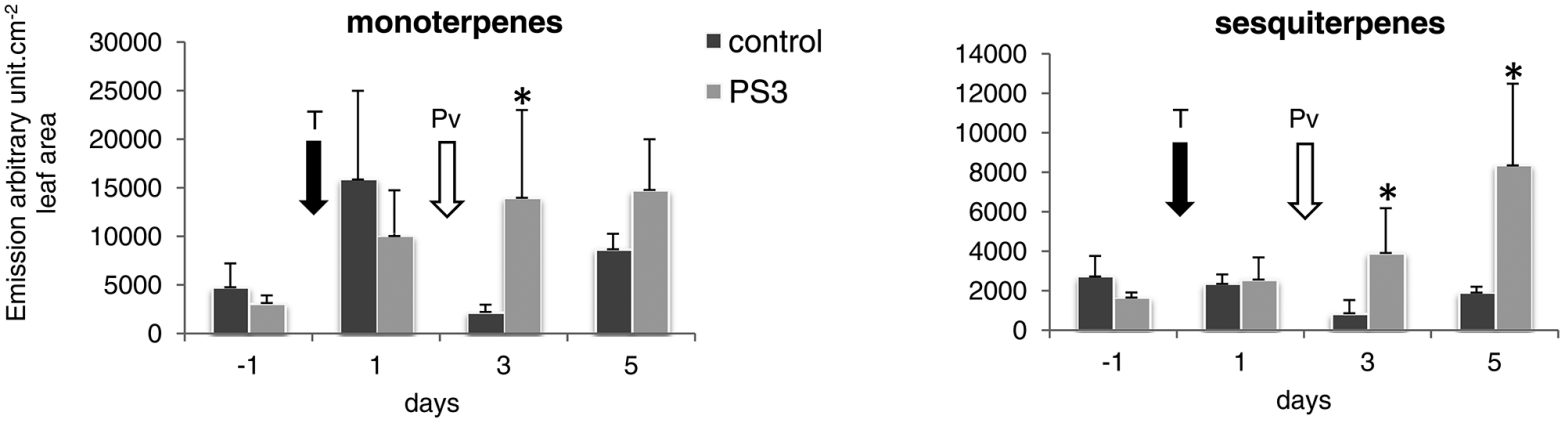
**Time-course study (SPME-GC-MS) of mono- and sesquiterpene emissions in the water- or PS3-treated and *P. viticola-*inoculated grapevine plants.** Both the PS3-treated and control samples were inoculated with *P. viticola* at 2 dpt. The values are the means of the sum of the quantities of all of the detected compounds calculated from three independent experiments (total number of plants per treatment = 15), expressed as arbitrary unit per cm^2^ of leaf area. The error bars represent the SDs. The arrows indicate the treatment and inoculation times. T, water or PS3 treatment; Pv, inoculation with *P. viticola*. Significant difference between the control and PS3-treated plants (*t*-test, ^∗^*p* < 0.05).

The VOC analyses at early times post treatment (6 hpt) did not reveal any qualitative nor quantitative significant variation compared with the control (not shown). As indicated in **Figure 4**, the overall monoterpene emissions increased sharply in the control 1 dpt, presumably as a response to spraying and enclosure in the bag. The monoterpene emissions then decreased and increased again at 5 dpt. In the PS3-treated samples, the VOC concentration in the bags increased from 1 to 5 dpt. At 3 and 5 dpt, the monoterpene emissions of the treated samples were higher than those of the control, although this difference was only significant at 3 dpt.

The emission of sesquiterpenes in the control remained steady throughout the experiment, regardless of inoculation, and increased significantly from 3 to 5 dpt in the PS3-treated samples.

**FIGURE 5:**
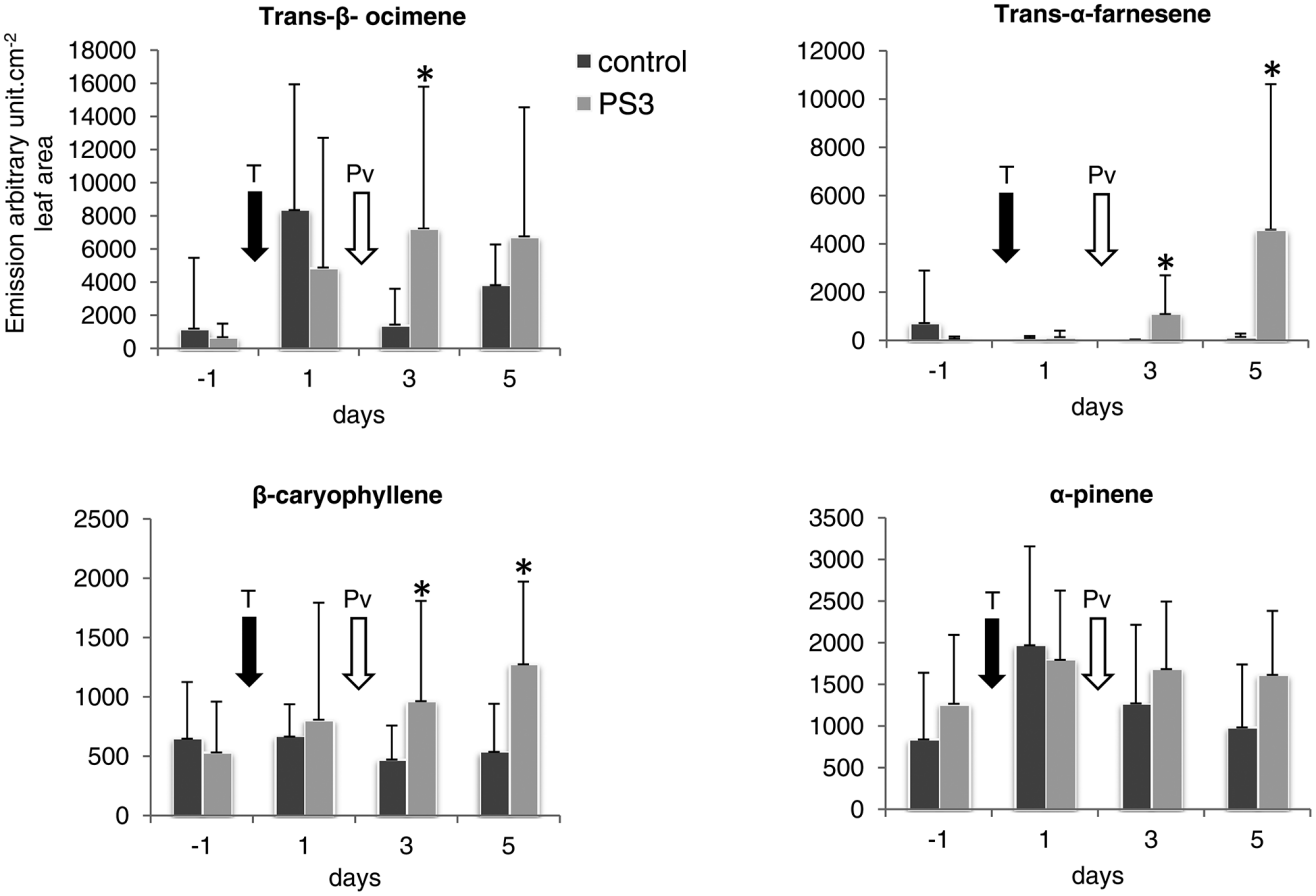
**Time-course study of the emission of representative VOCs in the water- or PS3-treated and inoculated grapevine plants.** The values are the means calculated from three independent experiments (total number of plants = 15), with the exception of 5 dpt, when only two experiments were performed (*n* = 10). The values are expressed as arbitrary units per cm^2^ of leaf area. The error bars represent the SDs. The arrows indicate the treatment and inoculation times. T, water or PS3 treatment, Pv, inoculation with *P. viticola*. Significant difference between the control and PS3-treated plants (*t*-test, ^∗^*p* < 0.05).

Among the identified terpenes, *trans*-β-ocimene and *(E,E)*-α-farnesene were the most abundant mono- and sesquiterpenes, respectively, produced by plants in response to PS3 (**Figure 5**). Although quite variable, *trans-β-*ocimene production was significantly higher in the PS3-treated samples 3 dpt. Interestingly, *(E,E)-*α**-**farnesene was hardly detectable in the control at any time and started to increase significantly from 3 to 5 dpt in the treated samples. The β-caryophyllene profile is similar to that of *(E,E)*-α-farnesene, although this compound was present in lower amounts.

**FIGURE 6:**
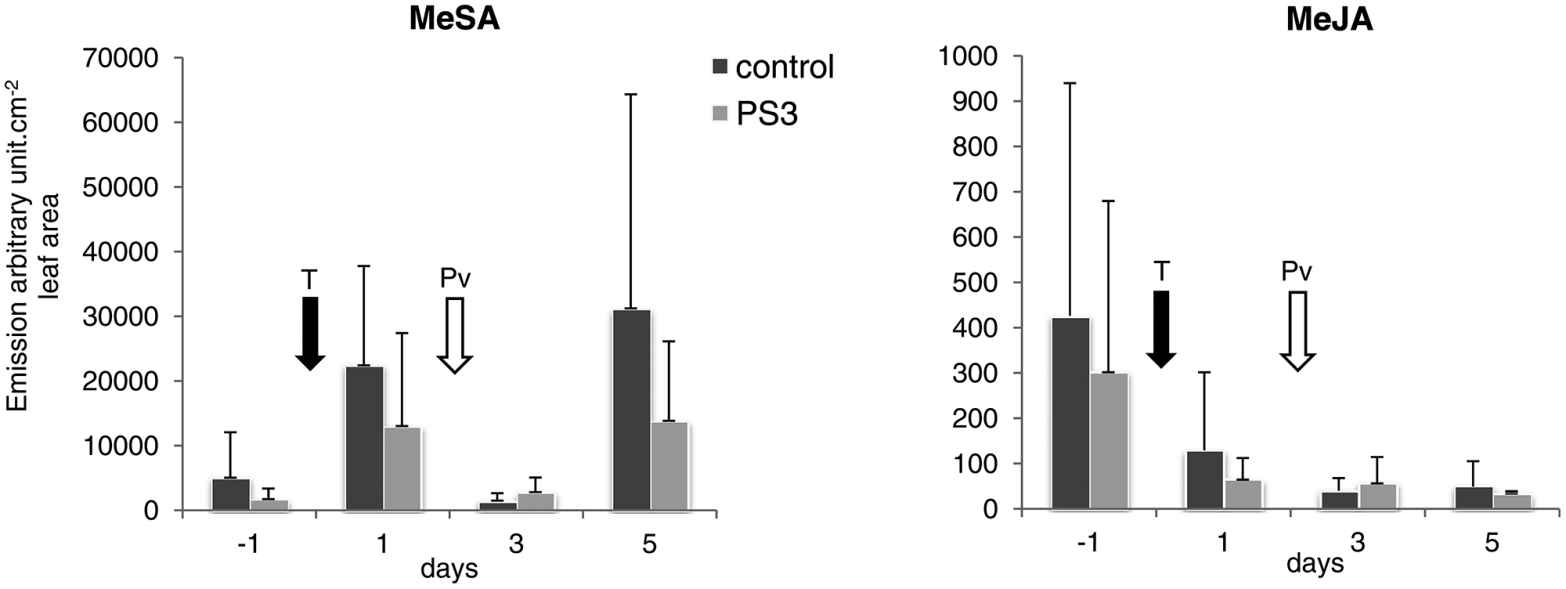
**Time-course study of the emission of MeSA and MeJA in the water- or PS3-treated and inoculated grapevine plants.** The values are the means calculated from three independent experiments (total number of plants = 15), with the exception of 5 dpt, when only two experiments were performed (*n* = 10). The values are expressed as arbitrary units per cm^2^ of leaf area. The error bars represent the SDs. The arrows indicate the treatment and inoculation times. T, water or PS3 treatment; Pv, inoculation with *P. viticola*.

Certain terpene compounds, e.g., α-pinene, remained remarkably steady in the control and PS3-treated samples. Similar to monoterpenes, the MeSA emissions peaked at 1 dpt, most likely as a response to the bagging procedure, and rapidly decreased in the control and PS3-treated plants (**Figure 6**). The MeSA emissions of the control and PS3-treated samples did not differ significantly during the time course of the experiment. However, in the control samples, the MeSA emissions increased significantly following inoculation (with a significant difference between 3 and 5 dpt, *p* < 0.01), confirming the kinetics of the production of this compound observed using PTR-QMS.

MeJA was detected in low amounts and did not appear to be affected by either PS3 treatment or fungal infection (data not shown). Of importance, the SPME-GC-MS results are in good concordance with the PTR-QMS results, strengthening our conclusion.

The PS3-enhanced emission rates of VOCs involve terpenes that are commonly observed in other plants. *Trans-β–*ocimene and *(E,E)*-α-farnesene, which are found to be among the more abundant terpenes emitted under our conditions, are also known to be products that are commonly emitted during plant-pathogen interactions (Jansen et al., 2011), supporting the concept that elicitors can mimic pathogen attack. This increased production of volatile terpenes is in agreement with the results of previous works that reported VOC emissions following the application of elicitors in other plant species. For instance, Obara et al. (2002) found that various elicitors, including a chitosan oligosaccharide, stimulates the emissions of GLV, MeSA, and mono- and sesquiterpenes in rice, with quantitative, and qualitative differences according to the treatment, whereas the rice blast fungus *Magnaporthe grisea* mainly induced the emission of mono- and sesquiterpenes and not MeSA. In line with this finding, other authors (Zhang and Chen, 2009) reported that chitosan treatment led to a strong induction of GLV and terpene emissions in tomato plants. Thus, it appears that the induction of VOCs is a common feature of elicitor treatment, including treatment with oligosaccharides, with differences according to treatment, plant species, and, likely, pathogens.

### Possible Roles of VOCs Emitted by Grapevine

As indicated in **Table 2**, PS3 treatment reduced the rate of disease in the three independent SPME experiments by 36–80%. The fact that PS3 protection is not statistically significant in Experiment 2 may be due to the absence of surfactant. In addition, variability in the infection rates also reflects the natural inconsistency of infection development inherent to these tests. In an attempt to establish a link between the VOC emissions from individual plants and their resistance to *P. viticola*, correlation analyses were performed (**Table 3**). Significant coefficients accounted for a negative correlation between the total amounts of terpenes in the control samples at 1 and 3 dpt (1 dpi) and the final infection rate at 8 dpt. This was also the case in the PS3-treated samples 3 dpt, which means that the plants with a lower infection rate at the end of the experiment, regardless of treatment, correspond to those that emitted more terpenes prior to (1 dpt) or at the beginning of the grape–pathogen interaction (3 dpt). Although care should be taken in interpreting correlations, early terpene production, regardless of whether it is constitutive or elicited, may play a role in grape defense. Later, an opposite correlation was found. The terpene production 5 dpt and the infection rate at 8 dpt were found to be positively correlated in the PS3-treated samples, an observation that could be interpreted as an enhanced capability of the elicited plants to produce terpenes in response to pathogen spread, although this reaction is not effective for reducing the disease.

**Table 2:** Induced resistance (IR) tests against *Plasmopara viticola* in SPME experiments.

Experiment		Disease severity
1	Control	58.2
	PS3	37.1^∗^
2	Control	15.7
	PS3	4.0
3	Control	17.5
	PS3	2.7^∗^

The disease severity values are the means of the sporulating leaf area percentages. Five plants were used for each condition. “^∗^”: significant difference (*p* < 0.03) between the control and PS3-treated samples.

**Table 3:** Correlation between VOC emission and infection rates (assessed 8 dpt).

	1 dpt		3 dpt (1 dpi)		5 dpt (3 dpi)	
	Control	PS3	Control	PS3	Control	PS3
Monoterp	-0.55^∗^	-0.26	-0.52^∗^	-0.59^∗^	-0.34	0.66^∗^
Sesquiterp	-0.62^∗^	-0.36	-0.70^∗^	-0.58^∗^	-0.49	0.74^∗^
MeSA	0,28	-0,16	-0,26	-0,45	0.66^∗^	0.83^∗^

The values correspond to Pearson’s coefficient calculated from the VOC emissions and sporulating foliar surface from all of the individual plants in the three greenhouse experiments through SPME-GC-MS analysis. The closer the coefficient is to 1, the stronger the correlation is. “^∗^” corresponds to a significant correlation (*p*-value <0.05), and “–” corresponds to a negative correlation.

It is well documented that volatile terpenes are part of the direct or indirect plant defense against pathogens (Kishimoto et al., 2006; Yi et al., 2009). For instance, β-caryophyllene, a sesquiterpene, contributes to the restriction of pathogenic *Pseudomonas* bacteria colonization in *Arabidopsis* (Huang et al., 2012). It is thus conceivable that various compounds, such as *(E,E)*-α-farnesene, in addition to the well-known stilbene derivatives, play a role in grape defense, but this requires further investigations. Moreover, it would be interesting to examine whether PS3-induced VOCs can act as plant-to-plant signals that induce pathogen resistance in neighboring plants, as demonstrated in the case of lima bean plants treated with the resistance inducer benzothiadiazole (BTH; Yi et al., 2009).

Methyl salicylate production at 3 dpi positively correlated with the infection rate in both the control and treated plants. This is consistent with increased emission of this compound observed with both methods of analysis during the course of the experiments in the inoculated plants. Even if longer time-course study could be useful to precise the kinetic of MeSA emissions, our results strongly suggest that they are linked to pathogen colonization. In this context, in contrast to the volatile terpenes, MeSA appears to act as a disease marker. This is similar to known cases of compatible interactions, in which phytoalexins are accumulated in response to pathogen infection but do not allow its restriction. These defense compounds should therefore be considered in these cases disease markers rather than resistance markers (Bailey and Deverall, 1971).

Grape VOCs constitutively emitted by reproductive organs serve as cues for grape moth oviposition. This insect is attracted by a ratio-specific volatile bouquet of common VOCs, including (*E,E*)-α-farnesene (Tasin et al., 2006). Altering the grape localization and oviposition by inducer treatments could be investigated in a future study.

In this study, sesquiterpenes, particularly (*E,E*)-α-farnesene, appear to represent a specific response to PS3 treatment. They could thus be considered possible biomarkers of the grapevine response to PS3 treatment. Further studies are needed to prove whether this is also true for other elicitors and to functionally link sesquiterpene emissions to IR against *P. viticola* and other pathogens.

To date, the use of elicitors, including PS3, suffers from an inconsistent level of disease control, and this is the major impediment to their practical application in the vineyard. IR relies on plant defense elicitation instead of the directly killing of the pathogen or insect, as is the case with pesticides, and it becomes clear that the full establishment of defense reactions may be conditioned by multiple factors, such as the development stage of plant organs, plant genotype, bioavailability of the elicitor in plant tissues, and interaction with abiotic factors (e.g., humidity, temperature, and water stress; Walters et al., 2013). In this context, reliable biomarkers of the plant response to elicitor treatments are needed to better understand the factors affecting IR. The monitoring of sesquiterpenes with GC-MS technique and SPME fibers, which is a rather convenient, non-destructive method, may be highly valuable for this purpose.

## Conflict of Interest Statement

The authors declare that the research was conducted in the absence of any commercial or financial relationships that could be construed as a potential conflict of interest.
